# Sleep duration and mortality among older adults in a 22-year follow-up study: an analysis of possible effect modifiers

**DOI:** 10.1007/s10433-014-0318-8

**Published:** 2014-06-12

**Authors:** Katarzyna Zawisza, Beata Tobiasz-Adamczyk, Aleksander Galas, Monika Brzyska

**Affiliations:** Chair of Epidemiology and Preventive Medicine, Jagiellonian University - Medical College, Kraków, Poland

**Keywords:** Older adults, Mortality, Sleep duration, Modification effect

## Abstract

The aim of this study was to assess the relationship of sleep duration and all-cause mortality among 2,449 Polish community-dwelling older citizens of Krakow observed during 22 years of follow-up. In particular, the role of some demographic, psychosocial and health-related conditions were investigated in terms of modification effect. In the prospective study, background information was gathered by face-to-face interview. Vital data were obtained from the population registry. Cox regression models were used to assess the role of sleep duration in mortality, in the analyses of potential effect modifiers and the shape of the relationship. Sleep duration was observed to be a significant predictor of all-cause mortality. Life-weariness, functional activity, total number of chronic diseases and age (65–79, 80+) were found to be effect modifiers for the relationship between sleep duration and mortality. Further investigation showed a U-shaped mortality risk associated with the duration of sleep among individuals with a high level of life-weariness, high functional activity and in individuals aged 80 and over. On the other hand, a linear relationship between longer sleep duration and mortality was observed among older people with no experience of life-weariness, without chronic diseases, with medium functional activity and aged 65–79, but also among those who reported three and more chronic conditions. Results of our study support available evidence showing the relationship between sleep duration and mortality among older adults and suggest that any public health intervention in this area should consider also other coexisting modifiable psychosocial and functional determinants.

## Introduction

Sleep, as a part of a healthy lifestyle, plays an important role in sustaining a high quality of life and good health (Faubel et al. 2009; Magee et al. 2011). There is a large body of scholarship showing the relationship between sleep duration and morbidity or mortality. It has been observed that either short or long habitual sleep is associated with hypertension (in different age groups including middle-aged and older individuals) (Gottlieb et al. 2006), with events of nonfatal and fatal cardiovascular disease (Elwood et al. 2006) and with depression (Patel et al. 2006). Additionally, extreme sleep duration in older adults was associated with lower health-related quality of life, also after adjustment for several covariates (Faubel et al. 2009).

Starting with one of the pioneer study in the topic, that is the Alameda study published by Kaplan (Kaplan et al. 1987), an increasing evidence that short or long sleep duration may be related to mortality risk has been observed (Burazeri et al. 2003; Cappuccio et al. 2010; Castro-Costa et al. 2011; Gallicchio and Kalesan 2009; Patel et al. 2004; Qiu et al. 2011). One comprehensive study suggested duration of sleep as an independent predictor of survival among older individuals from eight areas in Great Britain (Gale and Martyn 1998). Some studies showed that only long sleep (>8 h/day) was associated with high excess in mortality (Lan et al. 2007; Qureshi et al. 1997). Other results supported a U-shaped relationship between sleep duration and all-cause mortality (Kripke et al. 2002), but again another study showed this relationship only among adults aged 60–86 (Gangwisch et al. 2008) or only among those aged 50–59 (Kaplan et al. 1987). In contrast, some studies did not find any correlation (Ferrie et al. 2007).

Non-normative sleep duration may be related to several factors associated with increased mortality. It was observed that women who slept <6 or more than 7.5 hours per night if compared with women who slept 6.8–7.5 hours per night had higher odds of having a functional limitation (Goldman et al. 2007). Females with more disrupted sleep, as characterized by short sleep duration and longer awake time during the night, were at greater risk of poorer daily activities, and additionally, more fragmented sleep (as measured by minutes of wake after sleep onset) was associated with weaker neuromuscular performance and was a risk factor for functional impairment (Goldman et al. 2007). A Spanish study showed that women who had extreme sleep duration (either ≤5 h/day or ≥10 h/day) reported a worse score on the SF-36. The results of this study revealed also that long sleep duration was associated with poorer cognitive function in older adults from the general population (Faubel et al. 2009).

Although there is broad array of literature presenting the role of sleep in mortality, little is known about effect modifiers for this relationship. Gangwish and colleagues investigated whether age (ages 32–59 vs. 60–86 years) acted as an effect modifier in the relationship between short sleep duration (≤5 vs. 7 h) and mortality, and they found a significant interaction effect. In contrast, the interaction effect was not significant for long sleep duration (≥9 vs. 7 h) (Gangwisch et al. 2008). Similarly, it was observed that among the oldest old (meaning 80+ years), those who slept <7 hours or more than 9 hours had 18–21 % higher risks of mortality as compared to those who slept 8 hours. These results were not statistically significant in young elders (65–79 years) except for the category of 10 hours or more (Stamatakis et al. 2007). Another study performed among people aged 35+ did not support age as an effect modifier of the effects of sleep duration on all-cause mortality (Chien et al. 2010).

Considering gender, in a study in Mediterranean countries, long sleep duration was found to be a significant predictor of all-cause mortality among men aged 50 and over, whereas same-aged women were not affected (Burazeri et al. 2003). Analysis performed among Taiwanese older people (64+ years) showed a significant association between long sleep duration and mortality for both genders where the hazard ratio was higher among women (Lan et al. 2007). The Shirakawa study (Japan) showed that longer and shorter sleep controlled for several confounders (like present and past medical history, use of sleep pills, smoking and drinking habits) was significantly related to an increased risk of total mortality among males (Kojima et al. 2000). The study performed among 104,010 Japanese people aged from 40 to 79 years showed that sleep duration was a predictor of all-cause mortality among men and women (hazard ratios were slightly higher among females), but finally, in fully adjusted model, relation between short sleep duration and mortality was observed only among women (Tamakoshi and Ohno 2004). A later report of the Japanese cohort study showed a U-shaped relationship between sleep duration and all-cause mortality for both genders (Ikehara 2009). In one large study performed among people aged 30 and over in the USA, the results for males and females were also similar (Kripke et al. 2002), and the most recent critical review published by Kurina et al. (2013) has shown a lack of consistent differences between genders with regard to the association between sleep duration and mortality.

The association between sleep duration and all-cause mortality was shown to vary according to health status as assessed by a presence of preexisting illness and a functional limitation measured by MOS PF scale. Those who were healthy at baseline did not show the association, whereas among individuals in the less healthy group, higher risk of death was associated with short and long sleep duration. The authors additionally suggested that the aforementioned association might be the result of a residual confounding with poor baseline health (Magee et al. 2013).

There are also some other personal and environmental factors associated with the nature of sleep and related to mortality. “Sleep architecture” significantly changes with a number of psychosocial factors, such as the end of one’s career and the beginning of retirement or a death of a spouse, relatives or friends (Doghramji 2006; Ohayon 2004). Shorter sleep duration may also be the result of waking physical and psychological stressors. Alterations in the structure of sleep among older adults can result from previous occupational activity (like shift work), as well as from a number of stressful life events, which are common in older age. Low mood has been observed as significant determinant of quantity and quality of sleep in older age (Doghramji 2006).

Some epidemiological studies have shown that about 7–42 % of older adults report the presence of feelings related to prolonged fatigue—depending on the characteristics of the study population and the measurement tool used (Lewis and Wessely 1992; Prescott et al. 2003). There is also some evidence supporting the role of prolonged fatigue and tiredness in the development of chronic diseases (Ekmann et al. 2013) and mortality (Hardy and Studenski, 2008). Thus, both sleep duration and prolonged fatigue seem to be associated with morbidity and mortality; however, their interplay has not been investigated.

Additionally, work done by Garde and colleagues showed that perceived psychological pressure during work and leisure time was not a significant effect modifier for the association between sleep duration and ischemic heart disease mortality (Garde et al. 2013). Results of other studies suggested that SES, social and family support, and health practices did very little to modify the association between sleep duration and mortality (Mesas 2010; Stamatakis et al. 2007).

All in all, study results are somewhat inconsistent, and more importantly, data regarding possible effect modifiers for the relationship between duration of sleep and mortality, especially among older adults, are scarce. Thus, the aim of this study was to assess the relationship of sleep duration and all-cause mortality among 2,439 Polish community-dwelling older citizens of Krakow observed during 22 years of follow-up. In particular, the role of some demographic, psychosocial and health-related conditions was investigated in terms of modification effect.

## Methods

### Study design

**Fig. 1:**
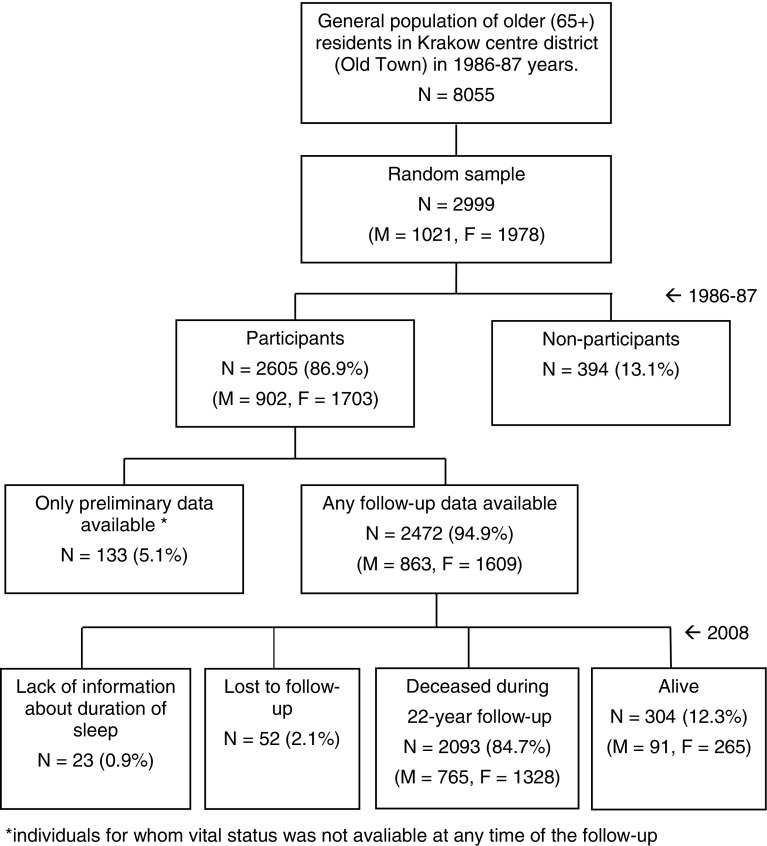
Study design

The detailed study design has been described elsewhere (Jedrychowski 1990; Tobiasz-Adamczyk et al. 2008). In brief, the baseline study evaluating the health status of older residents of Krakow city centre (south of Poland) was conducted in 1986–87. A group of 1,021 men and 1978 women was randomly sampled from a population of 8,055 noninstitutionalized residents (2,529 men and 5,526 women) of Krakow city centre, aged 65 and over. Vital status and the date of death were verified concurrently by the use of the national death register. 394 individuals (119 men and 275 women) were lost because of death or migration outside the study area before the interview or due to a refusal to participate. The final baseline sample (*n* = 2,605) represented 86.9 % of the original sample. From the 2,605 participants, 133 respondents were excluded because no information was available on their vital status at any time during the follow-up and 23 persons were also excluded due to missing data in the duration of sleep. Additionally, 52 individuals had been missing during the follow-up period. They were analyzed as ‘lost-to-follow-up’ (their final status remained unknown, although they contributed to the total person-time of the observation). The final sample available for the analysis did not vary significantly from the source population regarding basic demographic characteristic except age (final sample 72 years, drop outs 71 years, *p* = 0.001). The mean observation period in the cohort was 10.8 years (SD = 6.6), for a total of 26,415 person-years. A total of 2,093 deaths (765 men and 1,328 women) occurred until the end of 2008 (Fig. 1).

### Measures

At baseline, respondents were face-to-face interviewed using a structured questionnaire administered by trained interviewers. The interview included questions regarding demographic characteristics, health status, smoking history and daily activities. Self-reported average sleep duration was ascertained by the question: ‘‘How many hours of sleep do you get usually in a 24-h period?’’ An open-ended question was used, although the majority of respondents reported the time in 1-hour increments. Thus, the categories used in the analysis represent the center for the reporting time covering the period of 30 min around the value (e.g., 7 h ± 30 min). Next, for the descriptive part of the analysis, this variable was classified into 5 categories: 5 or fewer, 6, 7, 8, 9 or more hours of sleep.

The feeling of life-weariness was ascertained by the question expressing—in the native language—the long-lasting psychosocial condition considering several experiences like general life dissatisfaction, long-lasting physical and mental tiredness, loss of energy and general lack of “internal drive” regardless of its background (economical, psychosocial or medical). The wording of the question was: “Do you experience feelings of life-weariness?”, and respondents might answer “definitely no”, “yes, to a certain extent (sometimes)” or “definitely yes”. In the Polish language, the construct is similar in its meaning to prolonged fatigue or tiredness (but not depression). Although both ‘fatigue’ and ‘tiredness’ have their own native Polish names, they do not describe (in their colloquial usage) as much a generalized attitude to life.

Other information was also collected and considered as potential covariates. There were: (1) socio-demographic factors including gender, age (continuous variable, for the analysis of interaction considered as categorical <80 and 80+ years), education (primary or lower, vocational, high school and university degree); (2) the number of years of smoking ascertained by the question: “How many years have you smoked in total?”, where “never smokers” were indicated as 0; (3) the level of functional activity—created as a sum score reflecting participation in the following activities: (a) doing light household chores (Yes = 1; No = 0); (b) daily shopping (Yes = 3; No = 0), (c) doing heavy housework (Yes = 4; No = 0); (d) frequency of leaving home during a day and (e) the floor respondent lives on (from 0 to ground floor, at home all the time to 6.5—fifth floor (at least) and subject left their home at least three times a day due to different reasons including walking. The presented scoring system was prepared to achieve comparability with the ADL and IADL scores (Tobiasz-Adamczyk et al. 2005)—the continuous variable obtained in this way was categorized by tertiles into an ordinal variable with three categories: low, medium and high functional activity; (4) self-rated health status evaluated by the question: “How do you generally evaluate your health status?”—respondents might choose only one from five possible answers, ranging from very good through good, fair, poor to very poor. As the variable has shown relatively high correlation with the total number of diseases (gamma = 0.51; *p* < 0.001) and was observed to be redundant in the multivariable models it was used only in descriptive part; (5) to obtain a more precise picture of health status, respondents were also asked to indicate chronic diseases they were ever treated for (including asthma, emphysema, hypertension, cardiovascular disease and/or myocardial infarction, other heart disease, diabetes and chronic arthritis). If they answered “yes”, the respondent was considered as having the disease. Diseases were coded dichotomously. Finally, the total number of chronic conditions (categorized as 0, 1, 2, 3+) was considered as a covariate.

### Statistical analysis

The relations between self-reported average sleep duration and nominal variables were analyzed using the *χ* ^2^ test. Interval scale variables were tested for normal distribution by the Shapiro–Wilk test. When analyses failed to show normal distributions the Kruskal–Wallis test was used to test for significance.

The role of sleep duration (continuous), as well as gender, age (continuous), level of education (primary or lower, vocational, high school, university), functional activity (low, medium, high), total number of chronic diseases (0, 1 or 2, 3+), and number of years of smoking were assessed by Cox proportional hazard regression models. Firstly, univariate models were investigated. Next, the fully adjusted model was analyzed. Subsequently, the significance level for interaction between sleep duration and other covariates was tested. As published results suggest differences in the effect of sleep in mortality among younger (<80 years) and older (80+ years) (Qiu et al. 2011) the interaction term for age was investigated after such categorization. To verify a U-shaped relationship between sleep duration and mortality, a quadratic term of sleep duration was also added. Additionally, to limit the influence of sleep duration due to preexisting illness Cox regression models were performed after exclusion of subjects if deaths occurred within 2 years after entry. All statistical analyses were performed with IBM SPSS Statistics 21 for Windows.

## Results

Demographic and social as well as health characteristics of the baseline sample are presented in Table 1. The average age of men and women was nearly the same (72.0 (SD = 5.8), 72.5 (SD = 5.7), respectively). The average self-reported time of sleep was 7.6 (SD = 1.4) hours per day. A higher percentage of females was observed among those with short sleep duration (≤5 or 6 h/day). People who reported extreme sleep duration (≤5 or ≥9 h/day) compared with those who slept between 6 and 8 hours were slightly older, had a lower level of education and had worse functional activity, more frequently reported three or more chronic conditions and poorer self-rated health. Those who slept fewer hours more often reported the presence of feelings of life-weariness. A statistically significant difference was also found between sleep duration and number of years of smoking (see Table 1).

**Table 1:** Characteristics of population during the baseline study 1980–1987

	Categories of sleep duration					*p*
	≤5	6	7	8	≥9	
	*N* = 110	*N* = 540	*N* = 184	*N* = 1,310	*N* = 305	
Age (years)^a^	73.3 ± 6.2	72.0 ± 6.0	72.1 ± 6.0	72.3 ± 5.6	73.6 ± 5.9	0.007^b^
Females N(%)	79 (71.8)	376 (69.6)	105 (57.1)	835 (63.7)	198 (64.9)	df = 4; *p* = 0.010
Level of education *N* (%)						
Primary or lower	52 (47.3)	195 (36.2)	53 (28.8)	516 (39.4)	145 (47.5)	df = 12; *p* < 0.001
Vocational	12 (10.9)	54 (10.0)	18 (9.8)	147 (11.2)	45 (14.8)	
High school	26 (23.6)	197 (36.5)	57 (31.0)	419 (32.0)	79 (25.9)	
University	20 (18.2)	93 (17.3)	56 (30.4)	227 (17.3)	36 (11.8)	
Feelings of life-weariness *N* (%)						
Definitely no	32 (29.1)	131 (24.3)	126 (68.5)	441 (33.8)	184 (60.7)	df = 8; *p* < 0.001
Sometimes	71 (64.5)	372 (69.1)	47 (25.5)	828 (63.5)	104 (34.3)	
Definitely yes	7 (6.4)	35 (6.5)	11 (6.0)	35 (2.7)	15 (5.0)	
Functional activity *N* (%)						
Low	44 (28.4)	201 (17.1)	50 (23.7)	470 (15.6)	102 (32.7)	df = 8; *p* < 0.001
Medium	26 (36.7)	200 (51.8)	32 (23.2)	426 (50.7)	52 (29.9)	
High	39 (34.9)	136 (31.1)	95 (53.1)	387 (33.7)	140 (37.4)	
Number of chronic diseases *N* (%)						
0	10 (9.6)	59 (11.0)	22 (12.4)	162 (12.5)	26 (8.7)	df = 12; *p* < 0.001
1^e^	23 (22.1)	124 (23.2)	51 (28.7)	355 (27.3)	44 (14.7)	
2^e^	21 (20.2)	177(33.1)	32 (18.0)	404 (31.1)	69 (23.0)	
3 and more	50 (48.1)	174 (32.6)	73 (41.0)	379 (29.2)	161 (53.7)	
Self-reported health status *N* (%)						
Very good	1 (0.9)	14 (2.6)	14 (7.6)	37 (2.8)	10 (3.3)	df = 16; *p* < 0.001^f^
Good	75 (68.2)	466 (86.5)	104 (56.5)	1,076 (82.5)	119 (39.0)	
Fair	19 (17.3)	47 (8.7)	51 (27.7)	145 (11.1)	119 (39.0)	
Poor	9 (8.2)	10 (1.9)	10 (5.4)	38 (2.9)	44 (14.4)	
Very poor	6 (5.5)	2 (0.4)	5 (2.7)	9 (0.7)	13 (4.3)	
Number of years of smoking^a^	28.2 ± 12.3	28.5 ± 11.1	34.2 ± 13.5	29.0 ± 11.0	33.2 ± 13.4	*p* < 0.001^d^

^a^Continuous variable—data are presented as mean ± SD

^b^Kruskal–Wallis, post hoc analysis, significance observed only between: 6 versus ≥9 h/day (*p* = 0.003); 8 versus ≥9 h/day (*p* = 0.030)

^c^Kruskal–Wallis, post hoc analysis, significance observed only between: 8 versus ≥9 h/day (*p* < 0.001); 8 versus 7 h/day (*p* < 0.001); 6 versus ≥9 h/day (*p* < 0.001); 6 versus 7 h/day (*p* < 0.001); ≤5 versus ≥9 h/day (*p* = 0.008); ≤5 versus 7 h/day (*p* = 0.002)

^d^Kruskal–Wallis, post hoc analysis: ≤5 versus ≥9 h/day (*p* = 0.004), ≤5 versus 7 h/day (*p* < 0.001); 6 versus ≥9 h/day (*p* < 0.001); 6 versus 7 h/day (*p* < 0.001); 8 versus ≥9 h/day (*p* < 0.001); 8 versus 7 h/day (*p* < 0.001)

^e^For the further analyzes due to smaller sample sizes in some subgroups these categories were combined

^f^Fisher exact test was used

Cox regression analysis revealed a statistically significant increase in the risk of death associated with an increase in sleep duration (HR = 1.04). A lower risk of death was found for women (HR = 0.68) and for respondents with a university degree (HR = 0.78). In contrast, older age (HR = 1.08) and an increase in the number of years of smoking (HR = 1.008) increased the risk. Considering life-weariness, individuals reporting no feelings of life-weariness had about 10 % decreased risk of death as compared to the group reporting low level of life-weariness. Older people reported high functional activity had a 16–17 % decreased risk of death in comparison with those who reported low functional activity. Having more than three chronic diseases was associated with an about 35 % higher risk of death (see Table 2, Model 2).

**Table 2:** Risk (Hazards Ratios, HRs) of dying over the 22-year of follow-up across different characteristics

	Model 1		Model 2		Model 3	
	HR (95 % CI)	*p*	HR (95 % CI)	*p*	HR (95 % CI)	*p*
Sleep duration (hours)	1.06 (1.03–1.10)	<0.001	1.04 (1.01–1.07)	0.024	1.04 (1.003–1.075)	0.035
Gender (females vs. males)	0.73 (0.67–0.81)	<0.001	0.68 (0.61–0.75)	<0.001	0.71 (0.64–0.79)	<0.001
Age (years)	1.08 (1.07–1.09)	<0.001	1.08 (1.07–1.09)	<0.001	1.08 (1.07–1.09)	<0.001
Level of education: primary or lower	1		1		1	
Vocational	1.08 (0.93–1.24)	0.324	1.05 (0.91–1.22)	0.491	1.05 (0.86–1.19)	0.883
High school	0.91 (0.83–1.01)	0.082	0.95 (0.85–1.06)	0.340	0.96 (0.86–1.08)	0.516
University	0.87 (0.77–0.99)	0.032	0.78 (0.68–0.89)	<0.001	0.77 (0.66–0.89)	<0.001
No experience of life-weariness	1		1		1	
Low level of life-weariness	1.13 (1.03–1.24)	0.008	1.11 (1.01–1.23)	0.036	1.10 (0.99–1.22)	0.073
High level of life-weariness	1.12 (0.90–1.40)	0.298	1.20 (0.95–1.51)	0.202	1.16 (0.91–1.48)	0.227
Low functional activity	1		1		1	
Medium	0.90 (0.81–1.00)	0.042	1.01 (0.91–1.13)	0.846	1.01 (0.90–1.12)	0.962
High	0.71 (0.64–0.79)	<0.001	0.84 (0.75–0.94)	0.002	0.83 (0.74–0.94)	0.002
Number of chronic diseases: 0	1		1		1	
1–2	1.13 (0.97–1.32)	0.126	1.08 (0.93–1.25)	0.321	1.07 (0.92–1.25)	0.387
3 and more	1.36 (1.17–1.58)	<0.001	1.37 (1.18–1.60)	<0.001	1.34 (1.14–1.57)	<0.001
Number of years of smoking	1.007 (1.005–1.010)	<0.001	1.008 (1.005–1.010)	<0.001	1.007 (1.004–1.010)	<0.001

Model 1—unadjusted models

Model 2—adjusted for every variable presented in the table

Model 3—adjusted for every variable in the table. Analysis performed after exclusion of subjects if deaths occurred within 2 years after entry

Next, gender, age, level of education, number of years of smoking, life-weariness, functional activity and total number of chronic diseases were verified as potential effect modifiers for the relationship between sleep duration and mortality (Table 3). An interaction term was observed to be significant between age and sleep duration, life-weariness and sleep duration (between group with no experience of life-weariness and high level of life-weariness) suggesting that the effect of sleep duration depends on the level of life-weariness. Also, significant interaction effect was found between functional activity and sleep duration. Analysis performed after exclusion of subjects who died within 2 years after entry supported the results and, additionally, revealed significant interaction effect between sleep duration and number of chronic diseases (see Table 3).

**Table 3:** Hazards ratios for death associated with the interaction between sleep duration and potential effect modifiers: feelings of life-weariness, functional activity, total number of chronic diseases and age groups

Sleep duration (hours) interaction with:	Model 1		Model 2	
	HR (95 % CI)	p for interaction	HR (95 % CI)	p for interaction
Age (80 and over vs. 65–79 years)^a^	0.89 (0.81–0.98)	0.022	0.86 (0.77–0.96)	0.006
No experience of life-weariness^b^	1		1	
Low level of life-weariness	0.97 (0.91–1.04)	0.355	0.96 (0.89–1.03)	0.224
High level of life-weariness	0.83 (0.69–0.99)	0.040	0.79 (0.65–0.96)	0.016
Low functional activity^c^	1		1	
Medium functional activity	1.11 (1.03–1.20)	0.010	1.12 (1.02–1.21)	0.012
High functional activity	1.04 (0.96–1.12)	0.336	1.06 (0.98–1.15)	0.162
Without chronic conditions^d^	1		1	
One-two chronic condition	0.91 (0.81–1.01)	0.086	0.89 (0.79–0.998)	0.045
Three or more chronic conditions	0.97 (0.87–1.08)	0.564	0.94 (0.84–1.05)	0.284

^a^Adjusted for gender, level of education, functional activity, life-weariness, number of chronic diseases and number of years of smoking

^b^Adjusted for gender, age, level of education, functional activity, number of chronic diseases and number of years of smoking

^c^Adjusted for gender, age, level of education, life-weariness, number of chronic diseases and number of years of smoking

^d^Adjusted for gender, age, level of education, functional activity, life-weariness and number of years of smoking

Model 2 analysis performed after exclusion of subjects if deaths occurred within 2 years after entry

As the next step, we investigated the shape of mortality risk across different levels of life-weariness, functional activity, number of chronic diseases and age groups. As the risk estimates suggested a U-shaped association, two methods of data analysis using sleep duration as a continuous variable with: (1) linear (sleep duration) and (2) quadratic term (sleep duration squared) were performed.

The analysis showed a significant linear relationship for respondents aged from 65 to 79 at baseline and a U-shaped one for the oldest olds (80+). The expected U-shaped mortality risk associated with sleep duration was observed among individuals with high level of life-weariness, whereas among those who reported no experience of life-weariness the relation between sleep duration and mortality was linear. Similarly, a U-shaped relationship between sleep duration and mortality was shown for older people with high functional activity, while among those with medium functional activity only the linear component was significant. Analyses performed in subgroups considering the number of chronic diseases showed a linear relationship between sleep duration and mortality among people who did not reported any chronic condition as well as among these who reported three or more chronic diseases, suggesting that longer sleep duration was associated with increased mortality. Quadratic terms were insignificant for all analyzed subgroups of chronic diseases. The results of the aforementioned models for the whole sample as well as these performed after exclusion of respondents who died within 2 years after entry were similar, whereas for ages 80+ the omission of the first 2 years of follow-up results in nonsignificant linear and quadratic terms (see Table 4).

**Table 4:** Hazards Ratios for death associated with sleep duration by age, life-weariness, functional activity and number of chronic diseases (linear and quadratic models)

		Age^a^		Life-weariness^b^			Functional activity^c^			Number of chronic diseases^d^		
		65–79 years	80 and over	No experience	Low level	High level	Low	Medium	High	None	1–2	3 or more
Model 1a—linear model												
Sleep duration (hours) (Linear component)	HR 95 % CI	1.06 1.03–1.11	0.98 0.89–1.07	1.06 1.01–1.12	1.04 0.99–1.08	0.85 0.71–1.03	1.00 0.95–1.05	1.10 1.04–1.18	1.03 0.97–1.10	1.12 1.004–1.24	0.97 0.95–1.05	1.06 1.01–1.11
	p	0.001	0.669	0.023	0.117	0.099	0.934	0.002	0.277	0.042	0.871	0.013
Model 1b—quadratic model												
Sleep duration (hours) (Linear component)	HR 95 % CI	0.94 0.77–1.15	0.71 0.51–0.99	1.03 0.75–1.41	0.95 0.77–1.18	0.24 0.09–0.68	0.94 0.74–1.20	1.28 0.85–1.93	0.71 0.52–0.97	1.12 0.61–2.06	0.86 0.62–1.20	1.05 0.85–1.31
	p	0.552	0.045	0.850	0.666	0.007	0.601	0.248	0.030	0.720	0.376	0.640
Sleep duration (hours) (Quadratic component)	HR 95 % CI	1.01 0.995–1.02	1.02 1.001–1.39	1.00 0.98–1.02	1.01 0.99–1.02	1.09 1.02–1.16	1.00 0.99–1.02	0.99 0.96–1.02	1.02 1.01–1.05	1.00 0.96–1.04	1.01 0.99–1.03	1.00 0.99–1.01
	p	0.222	0.042	0.844	0.442	0.014	0.604	0.490	0.015	0.991	0.384	0.947
Model 2a—linear model												
Sleep duration (hours) (Linear component)	HR 95 % CI	1.07 1.03–1.11	0.95 0.86–1.06	1.07 1.01–1.13	1.03 0.99–1.08	0.83 0.67–1.02	0.99 0.94–1.05	1.10 1.03–1.18	1.05 0.98–1.11	1.14 1.02–1.27	1.00 0.95–1.50	1.06 1.003–1.11
	p	0.001	0.359	0.015	0.184	0.070	0.755	0.006	0.165	0.018	0.973	0.038
Model 2b—quadratic model												
Sleep duration (hours) (Linear component)	HR 95 % CI	0.92 0.75–1.14	0.76 0.37–1.59	1.00 0.72–1.38	1.06 0.77–1.46	0.16 0.06–0.47	1.00 0.77–1.58	1.20 0.79–1.84	0.68 0.49–0.94	1.21 0.63–2.34	0.85 0.60–1.20	1.06 0.78–1.44
	p	0.435	0.467	0.987	0.707	0.001	0.598	0.392	0.020	0.573	0.362	0.720
Sleep duration (hours) (Quadratic component)	HR 95 % CI	1.01 0.996–1.02	1.02 0.97–1.06	1.00 0.99–1.03	1.00 0.98–1.02	1.12 1.04–1.20	0.99 0.97–1.02	0.99 0.97–1.02	1.03 1.03–1.01	1.00 0.96–1.04	1.01 0.99–1.04	1.00 0.98–1.02
	p	0.159	0.546	0.661	0.854	0.002	0.561	0.669	0.008	0.860	0.359	0.976

^a^Models adjusted for gender, level of education, functional activity, life-weariness, number of chronic diseases and number of years of smoking; ^b^models adjusted for gender, age, level of education, functional activity, number of chronic diseases and number of years of smoking; ^c^models adjusted for gender, age, level of education, life-weariness, number of chronic diseases and number of years of smoking; ^d^models adjusted for gender, age, level of education, functional activity, life-weariness and number of years of smoking; Model 2a and 2b analysis performed after exclusion of subjects if deaths occurred within 2 years after entry

## Discussion

Our study showed an increase in the risk of dying associated with the duration of sleep, age, number of smoking years, number of chronic diseases, and a decrease in the risk among females, individuals with university level of education, and those with high functional activity, also after mutual statistical adjustment. Further analyses showed the presence of interaction between sleep duration and categories of age, experience of life-weariness, functional activity, and total number of chronic diseases. Finally, we observed differences in the shape of the relationship between sleep duration and mortality depending on the investigated characteristics of participants including different levels of functional activity and life-weariness. A linear relationship was suggested among elders with no experience of life-weariness, whereas in those with high level of life-weariness a U-shaped relationship was observed.

The linear relationship is in line with existing concepts explaining it by increased sleep fragmentation, feeling of lethargy observed in long sleepers or lack of physiologic challenge (Grandner and Drummond 2007). The point requiring elucidation is why we observed the difference in shapes for different levels of life-weariness. We propose that the increase in the risk of death among people experiencing high level of life-weariness is the effect of prolonged stress present in this group. Prolonged stress is one of well known physiological factors responsible for continuous activation of sympathetic nervous system and consequently for the development of some diseases, especially cardiovascular and subsequent increased risk of dying. If people are lacking coping strategies dealing with stress, they might have a short duration of sleep and this might be responsible for the U-shaped association observed in the ‘high level of life-weariness’ group. One should notice that at the time of data collection high level of social stress linked to the lack of financial and material resources, and low income, with limited access to goods was observed in Poland. Thus, the social environment of individuals under study was different that in any other study on sleep published to date. Additionally, some other social stressors, such as lack of social security, might contribute to the experience of life-weariness. These stressors might contribute to a very high risk of death among individuals with strong feelings of life-weariness and a very short sleep duration (sleep ≤5 h/day, HR = 9.04, 95 % CI = (2.55–32.12)).

Another modifying variable recognized in our study was functional activity. In the ‘medium functional activity’ group the sleep duration—mortality association was linear and in the ‘high functional activity’ a U-shape was observed. We observed no association between sleep duration and mortality among individuals in the ‘low functional activity’ group—this suggests the presence of some other unknown confounding variables (e.g., the presence of chronic condition as a determinant of low functional activity may confound the relationship and its very strong effect outweighs the role of sleep duration). It is not clear what are the underlying causes for the differences in shapes. One possible explanation is the aforementioned role of unmeasured stress. People who were still highly active (in our study we have measured a real performance, not an ability) had their own plans, aims to reach or things they had to do which might be the source of stress. Sleep deprivation itself is also a stressor and in some subgroups its consequences may be more clearly observed.

The sleep research literature also discusses the possibility of systematic response bias, as individuals with good health are more likely to report ‘normative’ values (meaning 7–8 hours of sleep). We believe that this type of bias is less likely in our study, as we observed the U-shaped association in the ‘high functional activity’ group. These are people who are more likely to have better general health. If the bias was present, we would observe no increase in the risk among people with lower duration of sleep. These people would report a higher than the real time of sleep duration and consequently the ‘short sleeper’ group would change in ‘the normal sleeper’ group resulting in a decrease in the risk among ‘short sleepers’ and in an increase in the risk of death in ‘normal sleepers’—but this was not observed.

Finally, our study has shown age as another possible effect modifier. We decided to verify the effect of a threshold of 80 years. This decision was driven by the results observed in published literature (Stamatakis et al. 2007). Individuals at age 80 and over presented a U-shaped association. This was not statistically significant after exclusion of subjects if death occurred within 2 years after entry. This does not necessarily contradict our results, as we argue that the significance was lost due to relatively short life expectancy in this group and a limitation of the sample size.

Our study has also some limitations: first, sleep duration might vary over the years of follow-up. As it was observed in other studies, however, for the majority of adults the time of habitual sleep remained unchanged, and a change in duration of >1 hour was observed in only 7 % of women and 4 % of men (Ferrie et al. 2011). Besides, feelings of life-weariness change with age, with the experiences of life events or with the change of economical or social status of a country (Ray et al. 1995). Functional activity changes too. In our study, data regarding variability over the follow-up were not available; subsequently, some unknown changes in time might distort our results. Second, all variables involved in the analysis were based on self-reported information; thus, we evaluate the subjective duration of sleep. There is evidence that subjective, self-reported sleep duration varies from sleep duration that is objective physiologically recorded (Lauderdale et al. 2008). Third, in our study we were not able to assess directly the validity and reliability of the life-weariness construct. We used the concept of “the feelings of life-weariness” which is very close to vital exhaustion characterized by unusual fatigue, loss of energy, increased irritability, and feelings of demoralization, assessed by the Maastricht Interview for Vital Exhaustion (Meesters and Appels 1996). There are also more related concepts like “feeling of tiredness” or “fatigue” used in the literature. To avoid misinterpretation we have decided to use the term “life-weariness”, which describes in the best way the expression used in our study and is very close but not the same as vital exhaustion. The use of one question only may also rise some concerns. Some other studies have shown, however, that single simple questions assessing general fatigue have very high test–retest reliability with interclass correlation of at least 0.9 (Hardy and Studenski 2008). Fourth, feelings of life-weariness are correlated with depression and both have much in common. We were unable to distinguish depression from feelings of life-weariness and to exclude the confounding effect of depression, depression-like disorders or low mood related to poor psychological and social well-being. Fifth, in our study we were also unable to remove the potential confounding effect of quality of sleep, taking sleep pills, or having a nap. Sixth, it is still open for discussion what the appropriate sleep duration is for older adults in Central Europe. We decided to use 7-hour sleep time as a reference period in our investigation. The same average sleep length of about 7 hours was observed in the investigation of community-dwelling elderly sample representative of the general population of seven European countries (Ohayon 2004), in the US Nurses’ Health Study (Burazeri et al. 2003) and in the NHANES (Gangwisch et al. 2008).

Although the presented study has some limitations, it provides a valuable contribution to the existing evidence. First, the study cohort consisted of a relatively large sample size of older adults living in the urban area, and it is the first such study performed in Central Europe considering people living in under specific social circumstances. In the analysis, we were able to control for most important covariates like gender, age, level of education, functional activity, and the number of chronic illnesses, as well as the number of years of smoking, which might confound an existing relationship. The presented results are also strengthened by a long follow-up and the fact that to make the observed relationships more likely, we excluded deaths which appeared in a short time after the beginning of the study, as they might be a result of some other underlying or subclinical conditions present at the time of recruitment. And finally, we provided evidence regarding possible effect modification by some important covariates, which is novel to our knowledge.

In summary, our study showed a relationship between the duration of reported habitual sleep and all-cause mortality among community-dwelling older individuals providing evidence regarding some parts of Central Europe. Modifying effects by the level of life-weariness, the level of functional activity, number of chronic conditions and age showed differences in the nature (either linear or U-shaped) of the association across these characteristics. Results of our study support available evidence showing the relationship between sleep duration and mortality among older adults and suggest that any public health intervention in this area should consider also other coexisting modifiable psychosocial and functional determinants.
